# Feasibility of in vivo ^18^F-florbetaben PET/MR imaging of human carotid amyloid-β

**DOI:** 10.1007/s00259-017-3651-2

**Published:** 2017-03-21

**Authors:** Jan Bucerius, Henryk Barthel, Solveig Tiepolt, Peter Werner, Judith C. Sluimer, Joachim E. Wildberger, Marianne Patt, Swen Hesse, Hermann-Josef Gertz, Erik A. L. Biessen, Felix M. Mottaghy, Osama Sabri

**Affiliations:** 1 Department of Radiology/Nuclear Medicine, Maastricht University Medical Center (MUMC+), Maastricht, The Netherlands; 2 Cardiovascular Research Institute Maastricht (CARIM), Maastricht University Medical Center (MUMC+), Maastricht, The Netherlands; 3 Department of Nuclear Medicine, University Hospital RWTH Aachen, Aachen, Germany; 4 Department of Nuclear Medicine/Radiology and Cardiovascular Research Institute Maastricht (CARIM), Maastricht University Medical Center (MUMC+), P. Debyelaan 25, 6229 HX Maastricht, The Netherlands; 5 Department of Nuclear Medicine, Leipzig University Medical Centre, Leipzig, Germany; 6 Department of Pathology, Experimental Vascular Pathology, Maastricht University Medical Center (MUMC+), Maastricht, The Netherlands; 7 Integrated Treatment and Research Centre (IFB) Adiposity Diseases, Leipzig University Medical Centre, Leipzig, Germany; 8 Department of Psychiatry, Leipzig University Medical Centre, Leipzig, Germany

**Keywords:** PET, Amyloid-β, Florbetaben, Atherosclerosis, Carotid arteries

## Abstract

**Purpose:**

Amyloid-beta (Aβ) peptides are involved in the inflammatory pathology of atherosclerosis. ^18^F-Florbetaben is a PET tracer for clinical imaging of cerebral Aβ plaques in Alzheimer’s disease (AD). We sought to determine whether specific uptake of ^18^F-florbetaben in the carotid arteries can be identified using a fully integrated hybrid PET/MRI system and whether this uptake is associated with clinical cardiovascular disease (CVD) risk factors.

**Methods:**

Carotid ^18^F-florbetaben uptake was quantified as the mean of the maximum target-to-background ratio (_mean_TBR_max_) in 40 cognitively impaired subjects (age 68.2 ± 9.5 years) undergoing ^18^F-florbetaben PET/MRI to diagnose AD. Associations between carotid ^18^F-florbetaben uptake and several CVD risk factors were assessed by univariate analysis followed by a multivariate linear regression analysis. Furthermore, carotid ^18^F-florbetaben uptake was compared between patients with and without a positive cerebral Aβ PET scan.

**Results:**

^18^F-Florbetaben uptake was clearly visualized in the carotid arteries. Values of _mean_TBR_max_ corrected for the blood pool activity of the tracer showed specific ^18^F-florbetaben uptake in the carotid wall. Male gender was associated with carotid ^18^F-florbetaben uptake in the univariate analysis, and was found to be an independent predictor of ^18^F-florbetaben uptake in the multivariate regression analysis (standardized regression coefficient *β* = 0.407, *p* = 0.009). Carotid ^18^F-florbetaben _mean_TBR_max_ in patients with a positive cerebral Aβ scan did not differ from that in patients without cerebral Aβ deposits.

**Conclusion:**

Specific ^18^F-florbetaben uptake in human carotid arteries was detected. Male gender was identified as an independent clinical risk factor. Therefore, ^18^F-florbetaben PET/MRI might provide new insights into the pathophysiological process in atherosclerosis.

## Introduction

The development of atherosclerotic plaques is characterized by accumulation of lipids, inflammatory cells and connective tissue within the arterial wall [1–3]. Even though atherosclerosis is usually a chronic, progressive process, abrupt rupture of an atherosclerotic – so-called vulnerable – plaque can become acutely life threatening by releasing embolic material that leads to myocardial infarction or stroke. So far, no biomarker or imaging technique is able to assess and predict the individual risk of plaque rupture and a subsequent acute cardiovascular event beforehand. Whether and how a plaque ruptures is determined by its macroscopic structure and its microscopic composition [4]. Arterial wall inflammation plays a key role in atherosclerotic plaque rupture [2]. Obviously, specific detection of markers of arterial inflammation within a vulnerable plaque through imaging represents a highly attractive approach to identifying patients at risk of rupture of such an atherosclerotic plaque [5].

Amyloid-beta (Aβ) deposition is considered one of the initial events in the pathogenesis of Alzheimer’s disease (AD). Furthermore, Aβ 1-42 peptides are involved in the inflammatory pathology of this neurodegenerative disorder [6, 7]. However, there is cumulative evidence that atherosclerotic disease as well as AD share some key pathophysiological elements and pathways that promote disease [6, 8, 9]. Irrespective of whether Aβ peptides are deposited in vessels of the central nervous system or atherosclerotic plaques in the periphery, they are likely to promote and perhaps synergize chronic inflammatory processes, which culminate in the degeneration, malfunction and vulnerability of arterial walls or plaques, respectively [6]. Activated platelets and/or vascular wall cells have been found to be the most likely source of the Aβ pool in aortic atherosclerosis lesions [6]. Aβ activates a cascade of proinflammatory events in endothelial cells and macrophages involving cytokine secretion and oxidative stress that leads to vascular disease [9–12]. In addition, it has recently been shown in blood samples from 1,464 haemodynamically stable patients that Aβ levels are significantly and independently associated with progression of arterial stiffness as well as with the incidence of subclinical atherosclerosis and coronary artery disease (CAD). Furthermore, Aβ blood levels can be used to substantially improve risk stratification for cardiovascular death in patients with stable CAD. [8].

Florbetaben is an ^18^F-labelled stilbene derivative that was developed as a PET tracer for routine clinical use to visualize Aβ plaques in the brain of AD patients [13]. A recently published multicentre phase 3 study revealed that ^18^F-florbetaben PET has high sensitivity and specificity for the detection of histopathologically confirmed neuritic Aβ plaques [14].

Using a fully integrated hybrid PET/MRI system, we evaluated whether specific ^18^F-florbetaben uptake in the carotid arteries can be identified and, subsequently, whether the carotid artery PET signal correlates with risk factors for clinical cardiovascular disease (CVD).

## Materials and methods

### Study design

This was a cross-sectional study of patients with suspected AD admitted to the Department of Nuclear Medicine, University of Leipzig, Germany, for further diagnostic evaluation with clinically indicated ^18^F-florbetaben PET/MRI. Scans were performed from May 2013 until August 2015 and were analysed for carotid ^18^F-florbetaben uptake. The analysis was approved by the institutional Ethics Committee and was performed according to the STROBE guidelines for cohort, case-control, and cross-sectional studies [15]. All subjects provided written informed consent for the PET/MRI examination.

### Questionnaire, biometric and biochemical measurements

The presence of CVD risk factors was prospectively assessed based on the results of a questionnaire retrieved from patients’ records. The presence of hypertension was defined as a history of systolic blood pressure >140 mm Hg, or a diastolic blood pressure >90 mm Hg. Diabetes was defined as documented diagnosis of type 1 or type 2 diabetic disease and the use of an antidiabetic treatment (diet, oral medication, insulin treatment). The definition of history of stroke included stroke and transitory ischaemic attack. Weight and height were measured and body mass index (BMI) calculated.

### ^18^F-Florbetaben PET/MRI

Simultaneous head/neck amyloid imaging with ^18^F-florbetaben was carried out on a fully integrated PET/MRI system (Biograph mMR; Siemens Healthcare, Erlangen, Germany). The PET data were acquired from 90 to 110 min after intravenous injection of 297.7 ± 9.37 MBq of the tracer. These data were reconstructed into a 256 × 256 matrix (voxel size 2.32 × 2.32 × 2.03 mm) using the built-in 3D ordered subsets expectation maximization algorithm with eight iterations, 21 subsets and a 3-mm gaussian filter. Standard corrections for decay, scatter, dead time and attenuation were performed. For attenuation correction, a two-point MRI Dixon VIBE sequence (TR 3.6 ms, TE 1.23 ms, slice thickness 3.12 mm, matrix 256 × 256, FOV 500 × 300 mm) was acquired at 3 T in parallel with the PET acquisition, resulting in segmented (air, soft tissue, fat) attenuation coefficient maps. Amongst other MR sequences, anatomical data via a 3D T1 magnetization-prepared rapid acquisition gradient echo (MPRAGE) sequence (TR 1,900 ms, TE 2.53 ms, slice thickness 1 mm, matrix 256 × 256, FOV 250 mm) were obtained in parallel with the PET acquisition.

### Image analysis

Images were analysed on a dedicated commercially available workstation (*syngo*.via VA30; mMR General Workflow; Siemens Healthcare). An experienced reader (J.B.) analysed all scans. The methodology for analysis and reproducibility of the vascular PET measurements has previously been reported [16]. Briefly, arterial ^18^F-florbetaben uptake was quantified by manually drawing an individual region of interest (ROI) around the left and right common carotid arteries on every slice of the T1-weighted MPRAGE scan. The ROIs were then used to acquire functional data from natively coregistered PET datasets. Next, the mean and maximum arterial standardized uptake values (SUV_mean_and SUV_max_; mean and highest pixel activity within the ROI) from PET were determined. Averaging the mean and maximum SUV values of all arterial slices of both carotid arteries led to _mean_SUV_mean_ and _mean_SUV_max_ values.

The mean and maximal carotid artery target-to-background ratios (TBR_mean_ and TBR_max_) were calculated by normalizing the carotid artery SUV_mean_and SUV_max_ to the mean SUV_mean_ of the blood pool measured in both jugular veins (JV). Thus, the carotid artery TBR is a reflection of the specific carotid artery wall ^18^F-florbetaben uptake. For measuring the JV SUVs, ROIs of 3–4 mm diameter were placed on consecutive slices bilaterally, and the results were averaged. TBR_mean_ and TBR_max_ were also averaged to obtain mean TBR_mean_ (_mean_TBR_mean_) and mean TBR_max_ (_mean_TBR_max_) values for both carotid arteries.

### Human atherosclerotic plaques and immunohistochemistry

A total of 40 atherosclerotic carotid arteries (38 patients, mean age 72 years, 64% men) were obtained at autopsy for the analysis of Aβ protein expression by immunohistochemistry in paraffin-embedded sections with intimal thickening, pathological thickening, a thick fibrous cap atheroma or intraplaque hemorrhage. All plaques evaluated were classified according to the method described by Virmani et al. [17] on slides stained with haematoxylin and eosin. Material was collected in agreement with the Dutch code of conduct for responsible use of human tissue (https://www.federa.org/code-goed-gebruik). All patient data has previously been reported [18]. For antigen retrieval, carotid artery sections and a AD-positive human brain section (positive control) were treated with 10 mM Tris/1 mM EDTA (pH 9) and then incubated overnight at room temperature with mouse anti-Aβ (DAKO M0172, diluted 1:50). Specific antigen–antibody binding was visualized by incubation with horseradish peroxidase-labelled goat anti-mouse secondary antibody (Brightvision) and diaminobenzidine.

### Statistical analysis

Continuous variables are expressed as means ± standard deviation and categorical data as absolute numbers and percentages. The normality of the data distributions was tested using the Kolmogorov-Smirnov test and differences were then evaluated using the *t* test for independent samples. Univariate analyses followed by a multivariate linear regression analysis with backward elimination were used to assess the associations between cardiovascular risk factors and carotid ^18^F-florbetaben uptake (_mean_TBR_max_) [19, 20]. ^18^F-florbetaben _mean_TBR_max_ was treated as the response (dependent) variable and cardiovascular risk factors as the explanatory (independent) variables for the regression analysis. The explanatory variables included were male gender, age >65 years, BMI, diabetes, hypertension, history of stroke, and history of CAD. Following this, the ENTER regression was used to determine independent predictors of the response variables. All explanatory variables in the backward elimination model found to be significantly associated with the ^18^F-florbetaben uptake value were retained and entered into the regression model as a block in a single step. This entry method was preferred over the forward selection of variables since only a few significant variables were left for a relatively low number of cases after excluding all the explanatory variables without a significant association with the different carotid wall ^18^F-florbetaben uptake values. The results of the multiregression models are presented with the standardized regression coefficients (*β*), the 95% confidence intervals, and the *p* values indicating the statistical significance of the estimates. All statistical analyses were performed using SPSS version 16.0 (SPSS, Chicago, IL).

## Results

### Aβ immunohistochemistry in human atherosclerosis

**Fig. 1:**
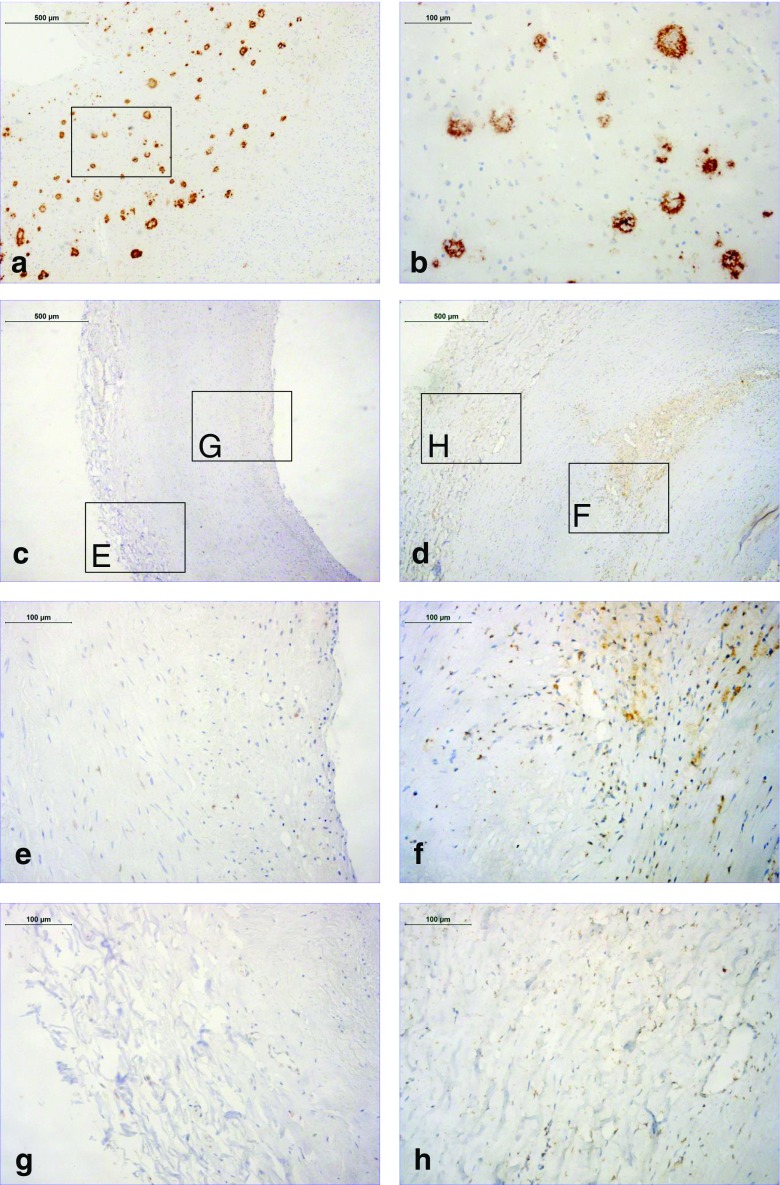
Aβ Immunohistochemistry in early and advanced human carotid plaques. **a**, **b** Aβ immunoreactivity (*brown*) in an AD-positive brain section (positive control tissue). The *inset box* in **a** is shown in more detail as the full image **b**. **c–h** Aβ immunoreactivity (*brown*) in carotid arteries from the 40 patients evaluated. The images show intimal thickening in an early plaque (**c**, **e**, **g**) and a thick fibrous cap atheroma in an advanced plaque (**d**, **f**, **h**). The *inset boxes E* and *F* and the same areas in more detail (full images **e** and **f**) show the plaque, and the *inset boxes G* and *H* and the same areas in more detail (full images **g** and **h**) show the adventitia

Aβ deposition was present in human atherosclerotic plaques (Fig. 1) as previously reported [21]. Aβ deposition was predominantly present in atherosclerotic plaques (Fig. 1e, f) and adventitia (Fig. 1g, h), but was absent from the media. In carotid arteries with intimal thickening only very few cells were positive (Fig. 1c, e, g), while clear deposition, mostly in macrophages, increasing with plaque progression to advanced atheromas (Fig. 1d, f, h).

### Aβ imaging in human carotid arteries

The PET/MRI scans in 40 patients provided appropriate imaging quality and were therefore suitable for analysis. Table 1 shows the demographic characteristics and ^18^F-florbetaben PET/MRI parameters of the study population. On average, 7 ± 4 slices of the left common carotid artery and 6 ± 4 slices of the right common carotid artery were analysed to derive the ^18^F-florbetaben uptake.

**Table 1:** Characteristics of the 40 included patients and their ^18^F-florbetaben PET/MRI data

Characteristic	Value
Age (years), mean ± SD	68.2 ± 9.5
Age >65 years, *n* (%)	26 (65.0)
Gender, *n* (%)	
Male	21 (52.5)
Female	19 (47.5)
Body mass index (kg/m^2^)	
Mean ± SD	25.8 ± 3.13
<25, *n* (%)	15 (37.5)
≥25 to <30, *n* (%)	21 (52.5)
≥30, *n* (%)	4 (10)
Medical history, *n* (%)	
Hypertension^a^	27 (73.0)
Diabetes^b^	7 (18.4)
Type I	2 (28.6)
Type II	5 (71.4)
History of stroke, *n* (%)^c^	2 (5.9)
History of coronary artery disease, *n* (%)^d^	5 (17.9)
Blood pool activity^e^	0.95 ± 0.22
Carotid uptake values^f^	
_ mean_SUV_mean_	1.07 ± 0.2
_ mean_SUV_max_	1.65 ± 0.34
Carotid TBR values^g^	
_ mean_TBR_mean_	1.15 ± 0.16
_ mean_TBR_max_	1.77 ± 0.38
Positive cerebral ^18^F-florbetaben PET/MRI, *n* (%)	21 (52.5)

*SUV* standardized uptake value, *TBR* target-to-background-ratio

^a^Three values missing

^b^Two values missing

^c^Twelve values missing

^d^Six values missing

^e^Average of the mean ^18^F-florbetaben SUV values of all analysed slices of the left and right jugular veins (_mean_SUV_mean_)

^f^By averaging the mean and maximum SUV values of all arterial slices of the left and right carotid artery, _mean_SUV_mean_ and _mean_SUV_max_ value were derived for the carotid arteries, respectively

^g^Carotid SUV_mean_ and SUV_max_ values divided by the blood pool _mean_SUV_mean_

**Fig. 2:**
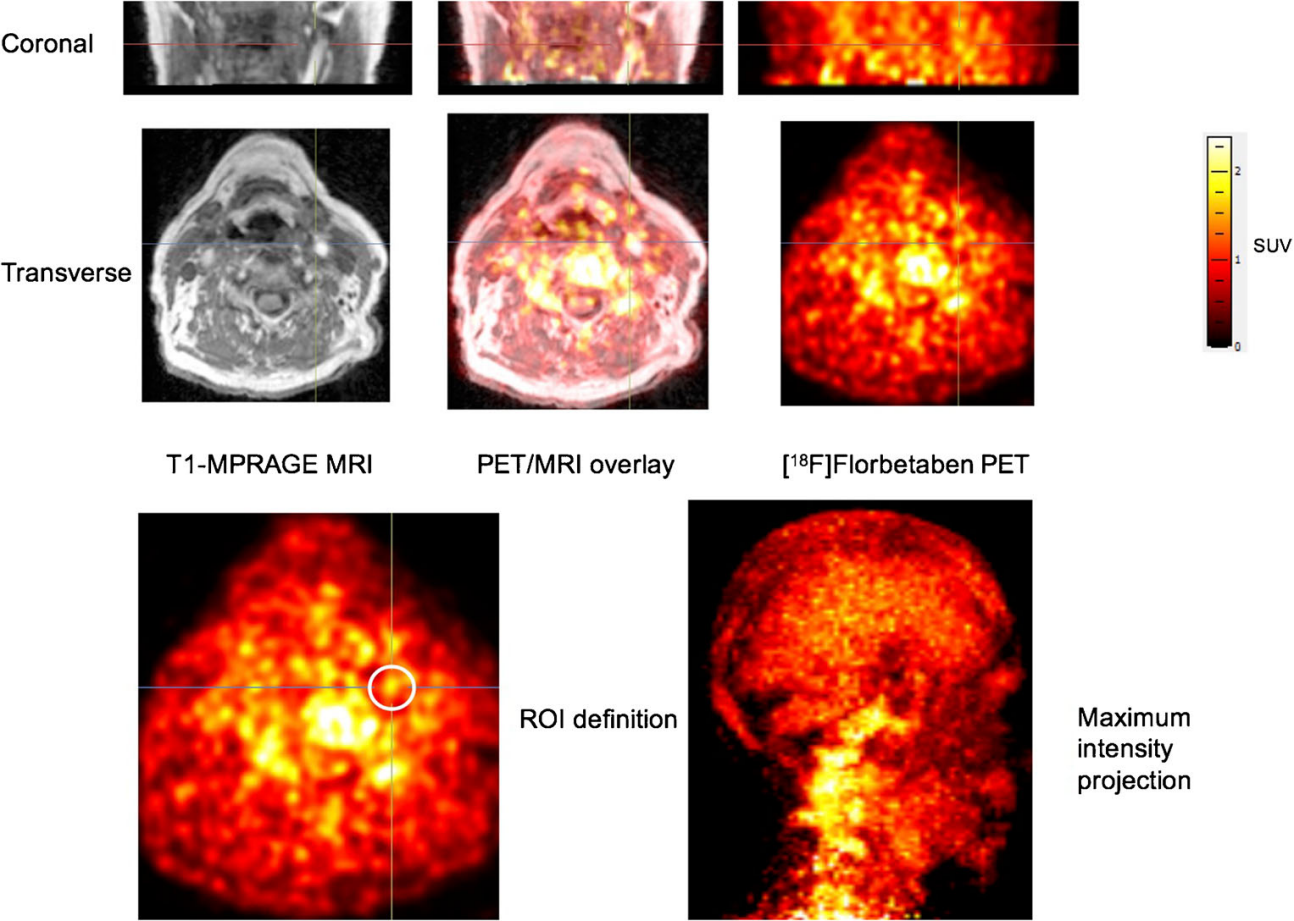
Focally increased ^18^F-florbetaben uptake in the left common carotid artery. *Top set of images* Coronal and transverse T1-W MR images, fused PET/MR images and PET-only images. *Bottom pair of images* Transverse PET slice with a ROI (*white circle*) placed around the lumen of the left common carotid artery including the focally increased ^18^F-florbetaben uptake, and a PET maximum intensity projection image with the carotid artery hot spot. Both visual and semiquantitative analyses revealed higher tracer uptake in the left than in the right common carotid artery (SUV_mean_ left 1.33, right 1.06; SUV_max_ left 1.83, right 1.22, ROI for the right common carotid artery not shown). A standard ROI within the lumen of the left jugular vein was used (not shown) for estimation of the ^18^F-florbetaben blood pool activity for calculation of the target-to-background ratio

In seven patients, the anatomy of only one common carotid artery (six left, one right) could be appropriately assessed. However, in all of these patients, analysis of the respective carotid artery provided sufficient data for inclusion in the analysis. ^18^F-florbetaben uptake in the carotid arteries as well as in the JV could be easily identified by visual inspection of the PET/MRI overlay images in all patients (Fig. 2). Furthermore, ROIs for semiquantitative analysis of ^18^F-florbetaben uptake were precisely placed in all analysed vessels.

The carotid _mean_SUV_mean_ and _mean_SUV_max_ values were significantly higher than the values of the venous blood pool (1.07 ± 0.2 vs. 0.95 ± 0.22 and 1.65 ± 0.34 vs. 0.95 ± 0.22, respectively; both *p* < 0.0001) leading to _mean_TBR_mean_ and _mean_TBR_max_ values >1 (Table 1). In 21 patients (52.5%), positive cerebral ^18^F-florbetaben uptake was seen. There was no significant difference in carotid ^18^F-florbetaben _mean_TBR_max_ between these patients and patients with a normal cerebral ^18^F-florbetaben scan (1.74 ± 0.44 vs. 1.81 ± 0.31, *p* = 0.536).

### ^18^F-Florbetaben and CVD risk factors

**Fig. 3:**
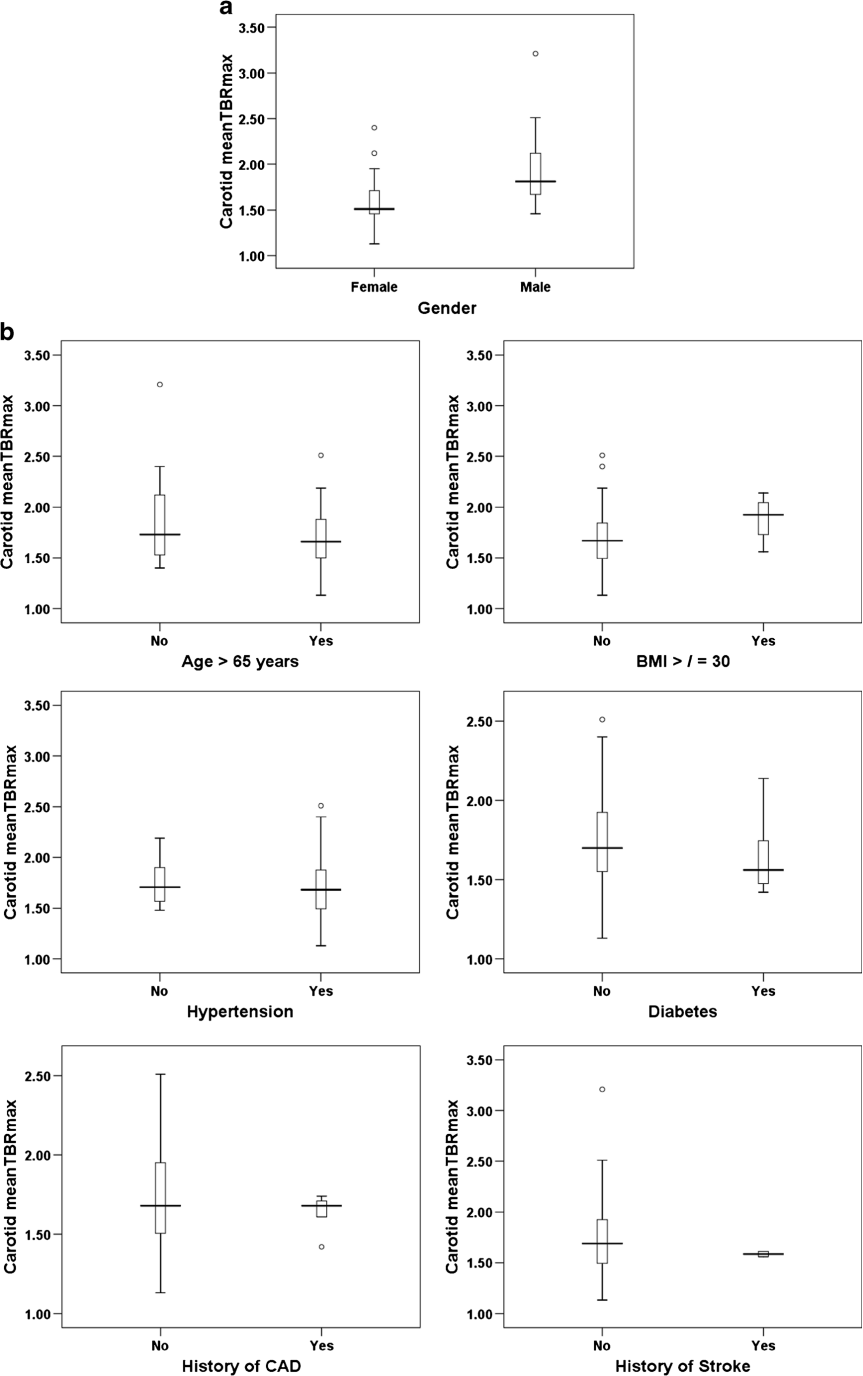
Relationships between cardiovascular disease risk factors and carotid ^18^F-florbetaben uptake expressed as _mean_TBR_max_. **a** Male gender was a significant independent predictor of carotid ^18^F-florbetaben uptake after correction for other risk factors (*p* = 0.009). Thus men showed significantly higher carotid _mean_TBR_max_ values than women. **b** Factors showing no statistically significant relationship with carotid ^18^F-florbetaben uptake: age >65 years, BMI ≥30 kg/m^2^, hypertension, diabetes, history of stroke, and history of coronary artery disease (CAD). None of these cardiovascular risk factors was significantly related to the ^18^F-florbetaben uptake in the carotid arteries in either the univariate analysis or the multivariate linear regression analysis. The data are presented as medians (*bold lines*), 25th and 75th percentiles (*boxes*), and 5th and 95th percentiles (*whiskers*); *circles* represent outliers

Table 2 shows the results of the univariate analysis of the association between carotid ^18^F-florbetaben uptake expressed as _mean_TBR_max_ and several well-known clinical CVD risk factors (male gender, age >65 years, BMI ≥30 kg/m^2^, diabetes, hypertension, history of stroke, history of CAD). Only male gender showed a significant association with the ^18^F-florbetaben uptake in the carotid arteries (1.92 ± 0.39 vs. 1.61 ± 0.30 in female patients, *p* = 0.009; Table 2, Fig. 3a, b). Multivariate linear regression analysis with backward elimination with a model including all variables as mentioned above was then performed to identify clinical CVD risk factors independently associated with carotid ^18^F-florbetaben uptake (Table 3). Male gender (*β* = 0.393, *p* = 0.038) was the only variable showing a significant association with the _mean_TBR_max_ in the carotid arteries. This risk factor (with a *p* value < 0.10) was exclusively retained in the model for the ENTER regression (Table 3). The ENTER regression showed that male gender was an independent positive predictor of ^18^F-florbetaben uptake (*β* = 0.407, *p* = 0.009; Table 2).

**Table 2:** Univariate analysis of the associations between clinical cardiovascular disease risk factors and carotid ^18^F-florbetaben uptake expressed as _mean_TBR_max_

Risk factor	95% confidence interval	*p* value
Male gender	0.08–0.527	0.009
Age >65 years	−0.412–0.09	0.202
Body mass index ≥30 kg/m^2^	−0.276–0.535	0.522
Diabetes	−0.366–0.146	0.389
Hypertension	−0.295–0.279	0.956
History of stroke	−0.779–0.397	0.513
History of coronary artery disease	−0.126–0.158	0.432

After confirming normality of the data distribution, univariate analysis was performed using the *t* test for independent samples

**Table 3:** Multivariate linear regression analysis with backward elimination and ENTER analysis to identify clinical cardiovascular disease risk factors associated with carotid ^18^F-florbetaben uptake expressed as _mean_TBR_max_ (the response variable)

	Explanatory variable	Standardized coefficient (*β*)	95% confidence interval	Adjusted *R* ^2^	Significance	*p* value
				0.122	0.038	
Backward analysis^a^	Male gender	0.393	0.014–0.477			0.038
				0.144	0.009	
ENTER analysis^b^	Male gender	0.407	0.08–0.527			0.009

^a^Explanatory variables: male gender, BMI ≥30 kg/m^2^, age >65 years, diabetes, hypertension, history of stroke, history of coronary artery disease. Variables were retained in the model when *p* < 0.10 and then entered into the ENTER analysis

^b^Explanatory variable: male gender

## Discussion

“Classical” imaging modalities such as invasive angiography, CT angiography and MRI do not provide sufficient information on specific characteristics (e.g. vulnerability) of arterial plaques. Identifying individual patients with vulnerable arterial plaques carrying a high risk of rupture is therefore a central challenge in cardiovascular medicine. Over the past decade there has been an increasing interest in noninvasive functional imaging of vascular inflammation and different parts of the underlying complex pathological processes of atherosclerosis by means of PET [22]. To our knowledge, amyloid tracers have not so far been utilized in this context. We identified PET imaging with the Aβ tracer ^18^F-florbetaben as a suitable approach to specifically identifying patients with increased carotid ^18^F-florbetaben uptake. The significantly higher arterial ^18^F-florbetaben uptake compared with the venous blood pool activity of the tracer clearly indicates specific arterial tracer uptake, which consequently led to _mean_TBR_mean_ and _mean_TBR_max_ ratios of >1. A nonspecific arterial tracer uptake would be expected to show a ratio of ≤1 [23]. Therefore, it is likely that ^18^F-florbetaben PET is able to specifically image Aβ peptides in the arterial wall and/or the atherosclerotic plaque even though histological confirmation of our imaging results are lacking. Our results indicate that ^18^F-florbetaben is able to image peripheral Aβ deposition in addition to the originally considered detection of intracerebral deposits in the clinic. In an approach comparable to ours, a similar PET tracer to ^18^F-florbetaben which is also used for noninvasive imaging of cerebral Aβ in AD, namely ^11^C-labelled Pittsburgh B compound (^11^C-PiB), was recently successfully used for noncerebral Aβ imaging in cardiac amyloidosis [24].

It is now considered that atherosclerosis and AD cerebrovascular amyloidosis share pathophysiological pathways, despite their different end-stage manifestations [6]. Lee et al. found that serum Aβ levels are elevated in stroke patients and that both the infarct size and the initial National Institutes of Health Stroke Scale (NIHSS) score are significantly positively correlated with serum Aβ levels [25]. More recently, the clinical relevance of Aβ with regard to CVD was further shown in a study by Stamatelopoulos et al. who found that Aβ blood levels in humans are independently associated with the progression of arterial stiffness, incident subclinical atherosclerosis, and incident coronary heart disease [9]. From a pathophysiological point of view, Kokjohn et al. found that atherosclerotic lesions contain a heterogeneous mixture of Aβ peptides [6]. These Aβ peptides most likely originate in the cells of the vascular walls that express Aβ precursor proteins/protease nexin-2/Aβ and in platelets that take part in the atherosclerotic inflammatory and disturbed coagulation cascades [6, 21, 26]. Both processes are inherent to arterial wall degeneration as part of the atherosclerotic disease process. The presence of Aβ peptides in atherosclerotic plaques may synergistically increase the chronic inflammatory processes that sustain this degeneration and the destruction of the arterial walls. This is because they mediate the process of increased and persistent synthesis of proinflammatory molecules by activated macrophages and activated platelets. As a consequence, focal vascular inflammatory response in the vulnerable region of atheroma plaques may contribute to plaque instability and rupture [27].

Although it appears that the general contributions of Aβ to the inflammatory reactions in atherosclerosis and AD are essentially the same [6, 13, 14], we did not observe higher carotid ^18^F-florbetaben uptake in patients with a positive cerebral Aβ scan than in those with a negative cerebral Aβ scan. Our results indicate that, despite the most likely shared pathophysiological basis of atherosclerosis and AD with regard to Aβ mediated inflammation, one cannot assume a completely common character of this inflammatory reaction in the brain and the non-cerebral arteries. This is in accordance with the results of the previously published Baltimore Longitudinal Study of Aging (BLSA), which showed that individuals with coronary or aortic atherosclerosis per se are not at increased risk of AD [28]. In contrast, intracranial atherosclerosis was confirmed as a strong risk factor for dementia [29]. However, our findings need to be confirmed in well-powered prospective trials.

Supporting the clinical relevance of our results, we found that male gender, one of the well-known CVD risk factors, was independently associated with ^18^F-florbetaben uptake in the carotid arteries after adjustment for other CVD risk factors. This is interesting as previous studies with ^18^F-FDG PET have also indicated a significant association between arterial ^18^F-FDG uptake, as a marker of arterial wall and/or plaque inflammation, and male gender [29–32]. Moreover, as revealed by our immunohistochemistry study, Aβ immunoreactivity was mostly present in atherosclerotic plaque macrophages. The fact that besides male gender none of the other evaluated CVD risk factors was found to be significantly related to carotid ^18^F-florbetaben uptake might be related to the rather small population in this study, the missing values for some of the collected cardiovascular risk factors and the fact that our patient population was not a dedicated cardiovascular risk population. Thus, well-powered, prospective studies need to be performed next to provide more conclusive results with regard to the association between arterial ^18^F-florbetaben uptake and clinical CVD risk factors.

Due to the study design involving the inclusion of patients with suspicion of AD, we were not able to confirm our imaging results by histology in these patients. Therefore, future studies need to histologically confirm the correlation between vascular ^18^F-florbetaben uptake and the presence of arterial Aβ deposition, in for example imaging of carotid artery stenosis. However, immunohistochemistry in a separate cohort representing different stages of atherosclerosis supported preferential uptake in advanced atherosclerotic plaques, while arteries with intimal thickening were mostly negative. Furthermore, in the absence of a dedicated vascular MR sequence in the retrospective setting of the study, we were not able to correlate the PET signal to a particular carotid plaque which might have been seen on the MRI scan. Another limitation was the rather small study population that potentially impaired the correlation between carotid ^18^F-florbetaben uptake and clinical CVD risk factors or distinct cardiovascular biomarkers as well as the correlation with the cerebral Aβ deposition. These issues will have to be addressed in future trials with dedicated CVD profiling.

### Conclusion

PET can visualize specific ^18^F-florbetaben uptake in human carotid arteries for which male gender was identified as an independent clinical risk factor. Therefore, we consider ^18^F-florbetaben PET/MRI as valuable for the noninvasive imaging of arterial Aβ deposition, providing new insights into the pathophysiological process of atherosclerosis.
